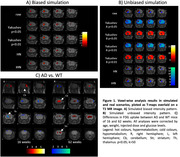# Optimizing PET Intensity Normalization for the Analysis of Cerebral Metabolism: A Study in Simulated and Real Data from an Alzheimer's Disease Model

**DOI:** 10.1002/alz.087454

**Published:** 2025-01-09

**Authors:** Helena Chamberlain‐Alvarado, Marta Cortes‐Canteli, Marta Casquero‐Veiga

**Affiliations:** ^1^ Universidad Carlos III de Madrid (UC3M), Leganés Spain; ^2^ Instituto de Investigación Sanitaria Fundación Jiménez Díaz (IIS‐FJD), Madrid Spain; ^3^ Spanish National Center for Cardiovascular Research (CNIC), Madrid Spain

## Abstract

**Background:**

Alzheimer's Disease (AD) has a long preclinical stage, in which brain metabolic alterations precede the symptoms onset. Therefore, an early and proper diagnosis of AD is essential for prevention and therapeutic evaluation. Current diagnosis and staging procedures rely on neuroimaging techniques, such as positron emission tomography (PET), which require intensity normalization to ensure the correct interpretation of the data. However, the variety of normalization methods and lack of standardization produce a major drawback regarding the proper interpretation of AD data by these techniques.

**Method:**

We formally analyzed the performance of six well‐established intensity normalization methods (proportional scaling and data‐driven approaches) on brain PET images of fluorodeoxyglucose (FDG) obtained from healthy mice (N=10). The accuracy of each method was determined based on the recovery of different computer‐simulated patterns in the FDG‐PET scans achieved with voxel‐wise analyses. Furthermore, we applied this newly acquired knowledge to evaluate differences in cerebral FDG uptake in 16 and 62 weeks‐old AD mice (N_16w_=7; N_62w_=10) of the TgCRND8 model, and their wild‐type littermates (N_16w_=10; N_62w_=5).

**Result:**

Our results showed a remarkable ambivalence between and within each normalization method under different simulation scenarios. They supported the use of an iterative histogram‐based normalization (iHN, Martí Fuster et al., 2013) when the data compared is not clearly biased (Figure 1B). Nonetheless, the reference cluster technique based on preserved regions (Yakushev et al., 2009) stood out for patterns with large and accentuated bias in the data (Figure 1A). Thus, the iHN method helped us to evaluate AD‐related differences in real FDG‐PET images, identifying genotypic effects that change with age (Figure 1C).

**Conclusion:**

This study evidences the crucial importance of understanding the distribution of the radiotracer uptake in PET studies before selecting an intensity normalization strategy. Furthermore, our results provide simple and accurate guidelines to help to choose a proper normalization method in preclinical PET imaging, demonstrating its applicability in real data from AD mice.

**References**:

Martí Fuster et al., 2013. Neuroinformatics 11(1), 77‐89.

Yakushev et al., 2009. Neuroimage 44(1), 43–50.

**Funding**: EU Joint Programme – Neurodegenerative Disease Research (JPND), BrightFocus Foundation, and JDC2022‐048922‐I funded by MCIN/AEI/10.13039/501100011033 and European Union NextGenerationEU/PRTR.